# Bis­(3-methyl-1-propyl-1,3-di­hydro-1*H*-imidazol-2-yl­idene)silver(I) chlor­ido(5,10,15,20-tetra­phenyl­porphin­ato)cadmate(II)

**DOI:** 10.1107/S2414314622004898

**Published:** 2022-05-17

**Authors:** J. P. J. Bruekers, J. A. A. W. Elemans, R. J. M. Nolte, P. Tinnemans

**Affiliations:** a Radboud University, Institute for Molecules and Materials, Heyendaalseweg 135, 6525 AJ, Nijmegen, The Netherlands; Goethe-Universität Frankfurt, Germany

**Keywords:** crystal structure, cadmium(II) porphyrin, silver(I)carbene

## Abstract

The structure of the title salt, [Ag(C_7_H_12_N_2_)_2_][CdCl(C_44_H_28_N_4_)], at 150 K has triclinic symmetry. One of the phenyl rings bonded to the porphyrin mol­ecule and the propyl groups of both yl­idene mol­ecules coordinating to silver are disordered over two positions.

## Abstract 2

The title compound consist of a cadmium(II) porphyrin with an axial chloride ligand and a silver(I) carbene.

## Graphical abstract

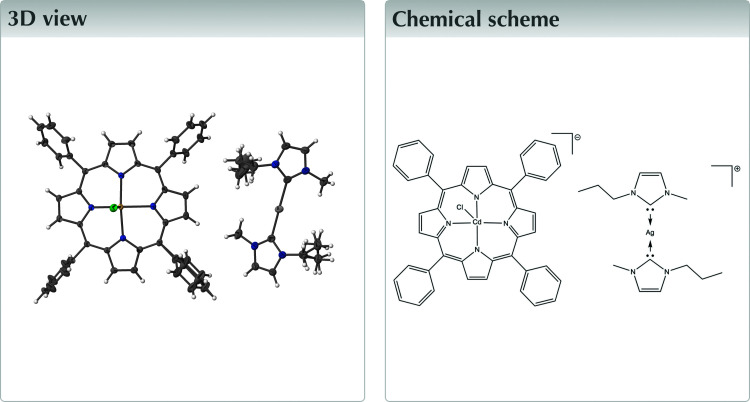

## Structure description

**Figure 1:**
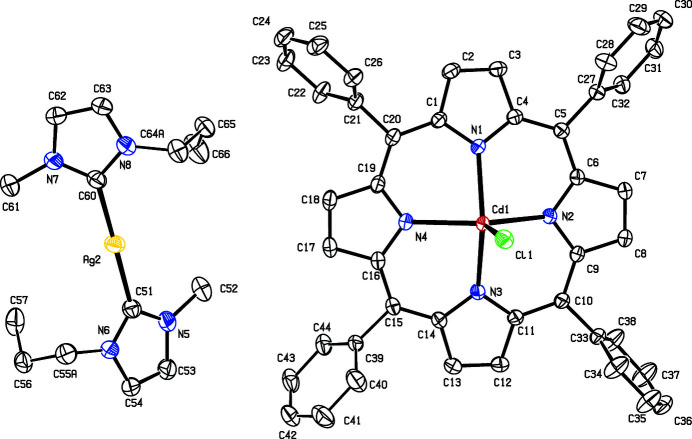
The structures of the mol­ecular entities in the title salt. Displacement ellipsoids are drawn at the 50% probability level. The minor-occupied sites of the disordered atoms have been omitted for clarity.

The structures of various cadmium(II) porphyrins have been determined previously. However, this exact combination of the chloride apical ligand and porphyrin structure has not been obtained before. In the absence of an apical ligand (Byrn *et al.* 1991 ▸), the cadmium porphyrin is planar and the cadmium atom resides in the mean plane through the porphyrin atoms. In the presence of an apical ligand like chloride (Zhang, Zhang, Wojtas *et al.*, 2012 ▸; Zhang, Gao *et al.*, 2012 ▸) or water (Toumi *et al.*, 2013 ▸), the cadmium centre is pulled 0.84–1.07 Å out of the mean porphyrin plane, which is similar to the distance in the title salt (Fig. 1 ▸) of 0.89 (12) Å. The dihedral angle between the imidazol-2-yl­idene planes is 41.49 (13)°, which is similar to the angle of 52.0° reported previously for a similar silver(I) carbene compound (Achar *et al.*, 2017 ▸). The angle between the Ag—C bonds in the title salt is close to linear at 179.14 (9)°, which is close to the angle of 170.6° reported by Achar *et al.* (2017 ▸).

**Figure 2:**
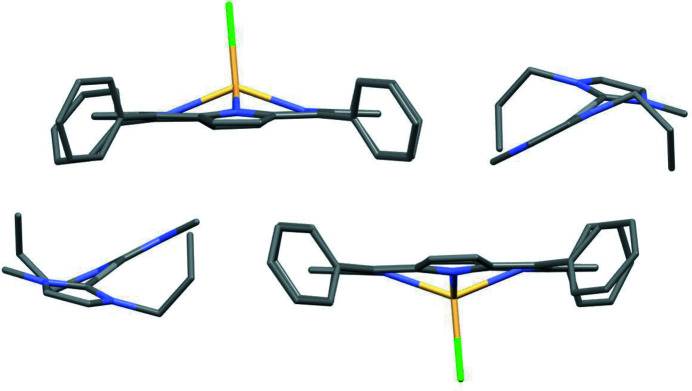
Two units of the stacked title salt, showing the offset stack alignment of the porphyrin planes relative to each other. H atoms and the minor-occupied sites of the disordered atoms have been omitted for clarity

The porphyrin planes show an offset stacked geometry (Fig. 2 ▸).

## Synthesis and crystallization

The title salt was obtained as a single crystal during an attempt to prepare and grow single crystals of a cadmium(II) tetra­phenyl­porphyrin carbene by mixing cadmium(II)–5,10,15,20-tetra­phenyl­porphyrin, 3-methyl-1-propyl­imidazolium chloride and silver(I) oxide in a 1:1:1 molar ratio in di­chloro­methane/*n*-heptane (1:1 *v*/*v*). The mixture was stored in the dark at 298 K, resulting in the formation of blue needle-shaped crystals after 72 h.

## Refinement

Crystal data, data collection and structure refinement details are summarized in Table 1 ▸. H atoms were placed in calculated positions and refined with a riding model, with *U*_iso_(H) = 1.5*U*_eq_(C) for methyl H atoms and 1.2*U*_eq_(C) otherwise. One of the phenyl rings of the porphyrin mol­ecule and the ethyl groups of both yl­idene mol­ecules coordinating to silver are disordered over two positions. The major conformation of the disordered phenyl ring has an occupancy of 0.703 (13). Bond lengths and angles of the minor fraction of the disorded benzene ring were restrained to be similar to those of the major-occupied fraction using the SAME instruction. The terminal ethyl groups of the propyl groups of both carbenes are disordered, with the occupancies of the major conformations being 0.679 (7) and 0.786 (11). The displacement parameters of bonded atoms were restrained to be similar using SIMU and RIGU instructions.

## Supplementary Material

Crystal structure: contains datablock(s) I, global. DOI: 10.1107/S2414314622004898/bt4123sup1.cif

Structure factors: contains datablock(s) I. DOI: 10.1107/S2414314622004898/bt4123Isup2.hkl

CCDC reference: 2171066

Additional supporting information:  crystallographic information; 3D view; checkCIF report

## Figures and Tables

**Table 1:** Experimental details

Crystal data	
Chemical formula	[Ag(C_7_H_12_N_2_)_2_][CdCl(C_44_H_28_N_4_)]
*M*_r_	1116.79
Crystal system, space group	Triclinic, *P* 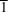
Temperature (K)	150
*a*, *b*, *c* (Å)	11.1004 (4), 13.1655 (5), 18.3723 (7)
α, β, γ (°)	84.0191 (17), 86.6707 (15), 70.0762 (14)
*V* (Å^3^)	2509.79 (16)
*Z*	2
Radiation type	Mo *K*α
μ (mm^−1^)	0.91
Crystal size (mm)	0.31 × 0.29 × 0.13

Data collection	
Diffractometer	Bruker D8 Quest APEX3
Absorption correction	Numerical (*SADABS*; Krause *et al.*, 2015 ▸)
*T*_min_, *T*_max_	0.811, 1.000
No. of measured, independent and observed [*I* > 2σ(*I*)] reflections	213430, 19237, 17410
*R*_int_	0.034
(sin θ/λ)_max_ (Å^−1^)	0.771

Refinement	
*R*[*F* ^2^ > 2σ(*F* ^2^)], *wR*(*F* ^2^), *S*	0.036, 0.108, 1.06
No. of reflections	19237
No. of parameters	721
No. of restraints	1420
H-atom treatment	H-atom parameters constrained
Δρ_max_, Δρ_min_ (e Å^−3^)	1.99, −2.60

Computer programs: *APEX3* (Bruker, 2017 ▸), *SAINT* (Bruker, 2003 ▸), *SHELXT2014* (Sheldrick, 2015*a* ▸), *SHELXL2018* (Sheldrick, 2015*b* ▸), *PLATON* (Spek, 2020 ▸) and *shelxle* (Hübschle *et al.*, 2011 ▸).

## full crystallographic data

### Crystal data

**Table:**

[Ag(C_7_H_12_N_2_)_2_][CdCl(C_44_H_28_N_4_)]	*Z* = 2
*M**_r_* = 1116.79	*F*(000) = 1136
Triclinic, *P*1	*D*_x_ = 1.478 Mg m^−^^3^
*a* = 11.1004 (4) Å	Mo *K*α radiation, λ = 0.71073 Å
*b* = 13.1655 (5) Å	Cell parameters from 9319 reflections
*c* = 18.3723 (7) Å	θ = 2.9–33.1°
α = 84.0191 (17)°	µ = 0.91 mm^−^^1^
β = 86.6707 (15)°	*T* = 150 K
γ = 70.0762 (14)°	Block, blue
*V* = 2509.79 (16) Å^3^	0.31 × 0.29 × 0.13 mm

### Data collection

**Table:**

Bruker D8 Quest APEX3 diffractometer	19237 independent reflections
Radiation source: sealed tube	17410 reflections with *I* > 2σ(*I*)
Graphite monochromator	*R*_int_ = 0.034
Detector resolution: 7.41 pixels mm^-1^	θ_max_ = 33.3°, θ_min_ = 2.1°
φ and ω scans	*h* = −17→17
Absorption correction: numerical (SADABS; Krause *et al.*, 2015)	*k* = −20→20
*T*_min_ = 0.811, *T*_max_ = 1.000	*l* = −28→28
213430 measured reflections	

### Refinement

**Table:**

Refinement on *F*^2^	Primary atom site location: structure-invariant direct methods
Least-squares matrix: full	Secondary atom site location: difference Fourier map
*R*[*F*^2^ > 2σ(*F*^2^)] = 0.036	Hydrogen site location: inferred from neighbouring sites
*wR*(*F*^2^) = 0.108	H-atom parameters constrained
*S* = 1.06	*w* = 1/[σ^2^(*F*_o_^2^) + (0.0492*P*)^2^ + 3.5802*P*] where *P* = (*F*_o_^2^ + 2*F*_c_^2^)/3
19237 reflections	(Δ/σ)_max_ = 0.005
721 parameters	Δρ_max_ = 1.99 e Å^−^^3^
1420 restraints	Δρ_min_ = −2.60 e Å^−^^3^

### Special details

**Table:**

Geometry. All esds (except the esd in the dihedral angle between two l.s. planes) are estimated using the full covariance matrix. The cell esds are taken into account individually in the estimation of esds in distances, angles and torsion angles; correlations between esds in cell parameters are only used when they are defined by crystal symmetry. An approximate (isotropic) treatment of cell esds is used for estimating esds involving l.s. planes.

### Fractional atomic coordinates and isotropic or equivalent isotropic displacement parameters (Å^2^)

**Table:**

	*x*	*y*	*z*	*U*_iso_*/*U*_eq_	Occ. (<1)
Cd1	0.42416 (2)	0.69550 (2)	0.71225 (2)	0.01906 (4)	
Cl1	0.55041 (5)	0.77535 (4)	0.78143 (3)	0.02852 (9)	
N1	0.24983 (15)	0.82356 (13)	0.66660 (8)	0.0214 (3)	
N2	0.27992 (15)	0.65635 (13)	0.79048 (9)	0.0220 (3)	
N3	0.51854 (16)	0.51550 (13)	0.72081 (9)	0.0223 (3)	
N4	0.48437 (16)	0.67998 (14)	0.59490 (8)	0.0242 (3)	
C1	0.24569 (18)	0.88289 (15)	0.60036 (10)	0.0227 (3)	
C2	0.12384 (19)	0.97021 (17)	0.59480 (11)	0.0281 (4)	
H2	0.096041	1.023256	0.554521	0.034*	
C3	0.05671 (19)	0.96205 (17)	0.65802 (12)	0.0279 (4)	
H3	−0.026842	1.008457	0.670278	0.033*	
C4	0.13636 (17)	0.86923 (15)	0.70338 (10)	0.0224 (3)	
C5	0.09997 (17)	0.83000 (15)	0.77306 (10)	0.0218 (3)	
C6	0.16882 (17)	0.73126 (15)	0.81346 (10)	0.0214 (3)	
C7	0.13225 (19)	0.69220 (17)	0.88490 (10)	0.0264 (3)	
H7	0.059414	0.728673	0.913683	0.032*	
C8	0.22172 (19)	0.59322 (17)	0.90336 (11)	0.0266 (3)	
H8	0.222797	0.547761	0.947187	0.032*	
C9	0.31451 (18)	0.57088 (15)	0.84341 (10)	0.0220 (3)	
C10	0.42052 (17)	0.47453 (14)	0.83970 (10)	0.0219 (3)	
C11	0.51128 (18)	0.44772 (14)	0.78126 (10)	0.0219 (3)	
C12	0.6092 (2)	0.34284 (16)	0.77501 (11)	0.0263 (3)	
H12	0.625068	0.280969	0.809445	0.032*	
C13	0.6740 (2)	0.34948 (16)	0.71070 (11)	0.0281 (4)	
H13	0.743640	0.293168	0.691602	0.034*	
C14	0.61667 (18)	0.45887 (15)	0.67670 (10)	0.0232 (3)	
C15	0.65374 (19)	0.49959 (16)	0.60806 (10)	0.0250 (3)	
C16	0.59096 (19)	0.60177 (16)	0.57051 (10)	0.0250 (3)	
C17	0.6272 (2)	0.63886 (19)	0.49831 (11)	0.0307 (4)	
H17	0.698553	0.600706	0.469122	0.037*	
C18	0.5403 (2)	0.73832 (18)	0.48013 (11)	0.0297 (4)	
H18	0.539124	0.782733	0.435789	0.036*	
C19	0.44944 (19)	0.76401 (16)	0.54134 (10)	0.0236 (3)	
C20	0.34025 (19)	0.85861 (16)	0.54385 (10)	0.0236 (3)	
C21	0.31851 (19)	0.93785 (16)	0.47740 (10)	0.0245 (3)	
C22	0.2886 (2)	0.9100 (2)	0.41127 (11)	0.0312 (4)	
H22	0.279346	0.841334	0.408858	0.037*	
C23	0.2721 (2)	0.9825 (2)	0.34867 (12)	0.0352 (5)	
H23	0.251782	0.962891	0.303810	0.042*	
C24	0.2852 (2)	1.0825 (2)	0.35148 (13)	0.0360 (5)	
H24	0.274342	1.131483	0.308613	0.043*	
C25	0.3142 (2)	1.1114 (2)	0.41687 (14)	0.0358 (5)	
H25	0.322973	1.180289	0.419000	0.043*	
C26	0.3305 (2)	1.03930 (18)	0.47953 (12)	0.0300 (4)	
H26	0.350123	1.059644	0.524319	0.036*	
C27	−0.02434 (18)	0.89793 (16)	0.80617 (10)	0.0234 (3)	
C28	−0.0439 (2)	1.00464 (18)	0.82000 (14)	0.0319 (4)	
H28	0.022507	1.034355	0.808452	0.038*	
C29	−0.1595 (2)	1.0684 (2)	0.85054 (15)	0.0379 (5)	
H29	−0.170638	1.140649	0.860055	0.046*	
C30	−0.2580 (2)	1.0272 (2)	0.86705 (14)	0.0385 (5)	
H30	−0.336711	1.070749	0.887798	0.046*	
C31	−0.2405 (2)	0.9222 (2)	0.85301 (13)	0.0347 (4)	
H31	−0.307945	0.893528	0.863727	0.042*	
C32	−0.12453 (19)	0.85777 (18)	0.82320 (11)	0.0274 (3)	
H32	−0.113749	0.785321	0.814379	0.033*	
C33	0.43734 (18)	0.38888 (15)	0.90240 (10)	0.0226 (3)	
C34	0.5279 (3)	0.3731 (2)	0.95519 (14)	0.0397 (5)	
H34	0.578828	0.418681	0.952273	0.048*	
C35	0.5455 (3)	0.2917 (2)	1.01234 (15)	0.0437 (6)	
H35	0.607545	0.282703	1.048190	0.052*	
C36	0.4739 (2)	0.22448 (19)	1.01725 (13)	0.0350 (4)	
H36	0.485747	0.169074	1.056381	0.042*	
C37	0.3847 (3)	0.2381 (2)	0.96481 (15)	0.0444 (6)	
H37	0.336226	0.190659	0.967048	0.053*	
C38	0.3649 (3)	0.3212 (2)	0.90847 (14)	0.0379 (5)	
H38	0.300713	0.331431	0.873732	0.045*	
C39	0.7664 (6)	0.4246 (11)	0.5690 (8)	0.0266 (11)	0.703 (13)
C40	0.8885 (4)	0.3961 (6)	0.5985 (3)	0.0500 (15)	0.703 (13)
H40	0.899224	0.426459	0.641337	0.060*	0.703 (13)
C41	0.9947 (5)	0.3230 (7)	0.5649 (3)	0.0631 (19)	0.703 (13)
H41	1.077755	0.305016	0.584177	0.076*	0.703 (13)
C42	0.9781 (8)	0.2773 (6)	0.5037 (5)	0.0490 (15)	0.703 (13)
H42	1.050146	0.228812	0.480216	0.059*	0.703 (13)
C43	0.8592 (6)	0.3012 (4)	0.4768 (3)	0.0370 (11)	0.703 (13)
H43	0.848613	0.268296	0.435128	0.044*	0.703 (13)
C44	0.7524 (6)	0.3737 (5)	0.5098 (3)	0.0290 (9)	0.703 (13)
H44	0.669510	0.388009	0.491285	0.035*	0.703 (13)
C45	0.7777 (16)	0.426 (3)	0.5760 (19)	0.030 (3)	0.297 (13)
C46	0.8887 (9)	0.4532 (15)	0.5746 (8)	0.050 (3)	0.297 (13)
H46	0.888475	0.516328	0.595476	0.060*	0.297 (13)
C47	1.0000 (9)	0.3861 (17)	0.5421 (10)	0.064 (4)	0.297 (13)
H47	1.077399	0.401519	0.543295	0.077*	0.297 (13)
C48	0.9993 (15)	0.2978 (18)	0.5082 (14)	0.051 (4)	0.297 (13)
H48	1.077187	0.247226	0.491606	0.061*	0.297 (13)
C49	0.8863 (14)	0.2841 (11)	0.4989 (9)	0.040 (3)	0.297 (13)
H49	0.883799	0.227406	0.471690	0.048*	0.297 (13)
C50	0.7716 (13)	0.3535 (10)	0.5293 (8)	0.030 (2)	0.297 (13)
H50	0.691105	0.349908	0.517396	0.036*	0.297 (13)
Ag2	0.90698 (2)	0.69473 (2)	0.19974 (2)	0.03222 (4)	
N5	0.9319 (2)	0.47300 (16)	0.28850 (11)	0.0327 (4)	
N6	1.10727 (19)	0.46983 (15)	0.23211 (10)	0.0296 (3)	
N7	0.8462 (2)	0.89703 (17)	0.08714 (11)	0.0347 (4)	
N8	0.7516 (2)	0.94014 (16)	0.18960 (11)	0.0339 (4)	
C51	0.9846 (2)	0.53447 (18)	0.24251 (12)	0.0299 (4)	
C52	0.7997 (3)	0.5085 (2)	0.31683 (16)	0.0436 (6)	
H52A	0.799411	0.510836	0.369982	0.065*	
H52B	0.758031	0.457415	0.305608	0.065*	
H52C	0.753102	0.580921	0.293897	0.065*	
C53	1.0214 (3)	0.3723 (2)	0.30666 (14)	0.0403 (5)	
H53	1.007855	0.314947	0.338064	0.048*	
C54	1.1317 (3)	0.37092 (19)	0.27135 (13)	0.0370 (5)	
H54	1.211037	0.312658	0.273296	0.044*	
C55A	1.2013 (2)	0.4974 (2)	0.18115 (14)	0.0355 (4)	0.679 (7)
H55A	1.284834	0.476254	0.205256	0.043*	0.679 (7)
H55B	1.171940	0.576699	0.167993	0.043*	0.679 (7)
C56	1.2179 (4)	0.4372 (3)	0.1102 (2)	0.0369 (9)	0.679 (7)
H56A	1.285406	0.453047	0.078216	0.044*	0.679 (7)
H56B	1.246697	0.357971	0.123815	0.044*	0.679 (7)
C57	1.0955 (4)	0.4699 (4)	0.0677 (2)	0.0460 (11)	0.679 (7)
H57A	1.028228	0.454223	0.098999	0.069*	0.679 (7)
H57B	1.110633	0.429061	0.024527	0.069*	0.679 (7)
H57C	1.068313	0.547779	0.052266	0.069*	0.679 (7)
C55B	1.2013 (2)	0.4974 (2)	0.18115 (14)	0.0355 (4)	0.321 (7)
H55C	1.208910	0.566394	0.193314	0.043*	0.321 (7)
H55D	1.286053	0.440193	0.188227	0.043*	0.321 (7)
C58	1.1682 (8)	0.5084 (6)	0.1047 (4)	0.0360 (17)	0.321 (7)
H58A	1.232833	0.530031	0.073635	0.043*	0.321 (7)
H58B	1.083771	0.565747	0.096845	0.043*	0.321 (7)
C59	1.1632 (8)	0.4017 (7)	0.0830 (4)	0.0375 (18)	0.321 (7)
H59A	1.241617	0.342881	0.098216	0.056*	0.321 (7)
H59B	1.155892	0.406156	0.029773	0.056*	0.321 (7)
H59C	1.088690	0.387368	0.107013	0.056*	0.321 (7)
C60	0.8317 (2)	0.85473 (18)	0.15588 (13)	0.0314 (4)	
C61	0.9308 (3)	0.8367 (3)	0.03077 (15)	0.0485 (7)	
H61A	0.887730	0.856493	−0.016301	0.073*	
H61B	0.951391	0.758683	0.044072	0.073*	
H61C	1.009948	0.854364	0.026752	0.073*	
C62	0.7750 (3)	1.0062 (2)	0.07772 (14)	0.0409 (5)	
H62	0.769901	1.053167	0.034218	0.049*	
C63	0.7144 (2)	1.0330 (2)	0.14213 (14)	0.0384 (5)	
H63	0.657282	1.102383	0.152815	0.046*	
C64A	0.7133 (3)	0.9366 (2)	0.26848 (15)	0.0384 (5)	0.787 (11)
H64A	0.716891	1.002339	0.288668	0.046*	0.787 (11)
H64B	0.775791	0.872783	0.294963	0.046*	0.787 (11)
C65	0.5796 (3)	0.9302 (3)	0.28222 (19)	0.0360 (8)	0.787 (11)
H65A	0.517840	0.992348	0.253920	0.043*	0.787 (11)
H65B	0.556495	0.937222	0.334773	0.043*	0.787 (11)
C66	0.5659 (5)	0.8245 (4)	0.2613 (3)	0.0526 (12)	0.787 (11)
H66A	0.581207	0.819865	0.208527	0.079*	0.787 (11)
H66B	0.479217	0.824132	0.274395	0.079*	0.787 (11)
H66C	0.628670	0.762285	0.287815	0.079*	0.787 (11)
C64B	0.7133 (3)	0.9366 (2)	0.26848 (15)	0.0384 (5)	0.213 (11)
H64C	0.787953	0.897820	0.299636	0.046*	0.213 (11)
H64D	0.670942	1.010409	0.284185	0.046*	0.213 (11)
C67	0.6288 (19)	0.8800 (18)	0.2704 (13)	0.077 (5)	0.213 (11)
H67A	0.685793	0.803314	0.271973	0.093*	0.213 (11)
H67B	0.588774	0.888877	0.319925	0.093*	0.213 (11)
C68	0.542 (3)	0.882 (4)	0.233 (2)	0.107 (9)	0.213 (11)
H68A	0.464223	0.940521	0.246321	0.160*	0.213 (11)
H68B	0.527391	0.812534	0.241062	0.160*	0.213 (11)
H68C	0.564434	0.895184	0.181331	0.160*	0.213 (11)

### Atomic displacement parameters (Å^2^)

**Table:**

	*U*^11^	*U*^22^	*U*^33^	*U*^12^	*U*^13^	*U*^23^
Cd1	0.02000 (6)	0.01896 (6)	0.01589 (5)	−0.00464 (4)	0.00228 (4)	0.00067 (4)
Cl1	0.0272 (2)	0.0300 (2)	0.0290 (2)	−0.00941 (17)	−0.00066 (16)	−0.00654 (17)
N1	0.0215 (6)	0.0222 (6)	0.0177 (6)	−0.0050 (5)	0.0002 (5)	0.0019 (5)
N2	0.0214 (6)	0.0205 (6)	0.0210 (6)	−0.0047 (5)	0.0036 (5)	0.0020 (5)
N3	0.0241 (7)	0.0198 (6)	0.0204 (6)	−0.0052 (5)	0.0042 (5)	−0.0010 (5)
N4	0.0258 (7)	0.0263 (7)	0.0169 (6)	−0.0049 (6)	0.0016 (5)	−0.0004 (5)
C1	0.0235 (8)	0.0238 (8)	0.0192 (7)	−0.0071 (6)	−0.0021 (6)	0.0032 (6)
C2	0.0247 (8)	0.0287 (9)	0.0263 (8)	−0.0057 (7)	−0.0040 (7)	0.0076 (7)
C3	0.0219 (8)	0.0274 (9)	0.0287 (9)	−0.0030 (7)	−0.0017 (6)	0.0045 (7)
C4	0.0202 (7)	0.0228 (7)	0.0221 (7)	−0.0054 (6)	−0.0002 (6)	0.0013 (6)
C5	0.0202 (7)	0.0215 (7)	0.0222 (7)	−0.0055 (6)	0.0010 (6)	−0.0012 (6)
C6	0.0214 (7)	0.0214 (7)	0.0199 (7)	−0.0062 (6)	0.0026 (6)	−0.0006 (5)
C7	0.0248 (8)	0.0290 (9)	0.0207 (7)	−0.0045 (7)	0.0057 (6)	−0.0001 (6)
C8	0.0263 (8)	0.0287 (9)	0.0207 (7)	−0.0063 (7)	0.0046 (6)	0.0028 (6)
C9	0.0225 (7)	0.0217 (7)	0.0201 (7)	−0.0072 (6)	0.0029 (6)	0.0021 (6)
C10	0.0219 (7)	0.0205 (7)	0.0217 (7)	−0.0067 (6)	0.0010 (6)	0.0021 (6)
C11	0.0222 (7)	0.0189 (7)	0.0228 (7)	−0.0055 (6)	0.0015 (6)	0.0002 (6)
C12	0.0267 (8)	0.0199 (7)	0.0280 (8)	−0.0034 (6)	0.0003 (7)	0.0003 (6)
C13	0.0288 (9)	0.0225 (8)	0.0277 (9)	−0.0018 (7)	0.0023 (7)	−0.0037 (6)
C14	0.0243 (8)	0.0218 (7)	0.0213 (7)	−0.0046 (6)	0.0028 (6)	−0.0042 (6)
C15	0.0253 (8)	0.0274 (8)	0.0197 (7)	−0.0052 (7)	0.0038 (6)	−0.0055 (6)
C16	0.0269 (8)	0.0285 (8)	0.0174 (7)	−0.0067 (7)	0.0036 (6)	−0.0030 (6)
C17	0.0342 (10)	0.0343 (10)	0.0207 (8)	−0.0088 (8)	0.0089 (7)	−0.0036 (7)
C18	0.0363 (10)	0.0329 (9)	0.0183 (7)	−0.0112 (8)	0.0061 (7)	−0.0001 (7)
C19	0.0271 (8)	0.0274 (8)	0.0160 (7)	−0.0096 (7)	0.0007 (6)	0.0005 (6)
C20	0.0271 (8)	0.0263 (8)	0.0173 (7)	−0.0102 (7)	−0.0016 (6)	0.0026 (6)
C21	0.0254 (8)	0.0295 (8)	0.0182 (7)	−0.0102 (7)	−0.0022 (6)	0.0045 (6)
C22	0.0382 (11)	0.0406 (11)	0.0190 (8)	−0.0204 (9)	−0.0067 (7)	0.0062 (7)
C23	0.0347 (10)	0.0508 (13)	0.0214 (8)	−0.0186 (10)	−0.0060 (7)	0.0084 (8)
C24	0.0317 (10)	0.0432 (12)	0.0303 (10)	−0.0141 (9)	−0.0042 (8)	0.0158 (9)
C25	0.0368 (11)	0.0321 (10)	0.0376 (11)	−0.0139 (9)	−0.0039 (9)	0.0106 (8)
C26	0.0328 (10)	0.0290 (9)	0.0273 (9)	−0.0106 (8)	−0.0040 (7)	0.0041 (7)
C27	0.0203 (7)	0.0248 (8)	0.0223 (7)	−0.0042 (6)	0.0002 (6)	−0.0016 (6)
C28	0.0251 (9)	0.0268 (9)	0.0407 (11)	−0.0037 (7)	−0.0007 (8)	−0.0065 (8)
C29	0.0317 (10)	0.0311 (10)	0.0431 (12)	0.0018 (8)	−0.0013 (9)	−0.0104 (9)
C30	0.0256 (9)	0.0441 (12)	0.0334 (11)	0.0042 (8)	0.0028 (8)	−0.0057 (9)
C31	0.0224 (9)	0.0460 (12)	0.0316 (10)	−0.0082 (8)	0.0054 (7)	−0.0011 (9)
C32	0.0245 (8)	0.0317 (9)	0.0244 (8)	−0.0082 (7)	0.0022 (6)	−0.0019 (7)
C33	0.0238 (8)	0.0215 (7)	0.0213 (7)	−0.0073 (6)	0.0001 (6)	0.0026 (6)
C34	0.0454 (13)	0.0411 (12)	0.0390 (12)	−0.0257 (11)	−0.0167 (10)	0.0147 (9)
C35	0.0510 (15)	0.0481 (14)	0.0356 (12)	−0.0243 (12)	−0.0191 (11)	0.0168 (10)
C36	0.0397 (11)	0.0319 (10)	0.0291 (10)	−0.0105 (9)	−0.0018 (8)	0.0111 (8)
C37	0.0559 (15)	0.0446 (13)	0.0418 (13)	−0.0332 (12)	−0.0145 (11)	0.0194 (11)
C38	0.0449 (12)	0.0418 (12)	0.0341 (11)	−0.0269 (10)	−0.0146 (9)	0.0148 (9)
C39	0.0227 (16)	0.033 (3)	0.018 (3)	−0.0007 (14)	−0.0005 (13)	−0.0039 (15)
C40	0.0305 (17)	0.075 (4)	0.0333 (19)	0.0015 (19)	−0.0013 (14)	−0.019 (2)
C41	0.033 (2)	0.086 (4)	0.046 (2)	0.012 (2)	0.0029 (17)	−0.014 (3)
C42	0.049 (3)	0.040 (3)	0.041 (3)	0.004 (2)	0.019 (3)	−0.0058 (18)
C43	0.053 (3)	0.0253 (19)	0.034 (2)	−0.0155 (19)	0.0191 (18)	−0.0101 (17)
C44	0.038 (2)	0.027 (2)	0.025 (2)	−0.0144 (16)	0.0078 (15)	−0.0068 (15)
C45	0.035 (5)	0.036 (6)	0.018 (6)	−0.009 (5)	0.006 (5)	−0.013 (5)
C46	0.030 (4)	0.077 (8)	0.047 (6)	−0.017 (4)	0.009 (4)	−0.038 (6)
C47	0.022 (4)	0.104 (10)	0.066 (8)	−0.009 (5)	0.005 (4)	−0.048 (7)
C48	0.028 (4)	0.065 (9)	0.049 (6)	0.000 (4)	0.014 (4)	−0.020 (6)
C49	0.043 (5)	0.030 (5)	0.042 (7)	−0.005 (4)	0.014 (4)	−0.009 (4)
C50	0.035 (4)	0.024 (4)	0.033 (6)	−0.011 (3)	0.011 (4)	−0.006 (4)
Ag2	0.03106 (8)	0.02611 (7)	0.03577 (8)	−0.00444 (6)	−0.00168 (6)	−0.00369 (6)
N5	0.0353 (9)	0.0324 (9)	0.0283 (8)	−0.0085 (7)	0.0053 (7)	−0.0072 (7)
N6	0.0317 (8)	0.0266 (8)	0.0276 (8)	−0.0058 (6)	−0.0001 (6)	−0.0032 (6)
N7	0.0344 (9)	0.0330 (9)	0.0288 (8)	0.0001 (7)	−0.0008 (7)	−0.0062 (7)
N8	0.0327 (9)	0.0298 (8)	0.0348 (9)	−0.0047 (7)	0.0042 (7)	−0.0056 (7)
C51	0.0319 (9)	0.0284 (9)	0.0283 (9)	−0.0074 (7)	−0.0014 (7)	−0.0062 (7)
C52	0.0404 (13)	0.0481 (14)	0.0411 (13)	−0.0127 (11)	0.0127 (10)	−0.0136 (11)
C53	0.0491 (14)	0.0311 (10)	0.0336 (11)	−0.0071 (9)	0.0101 (10)	0.0003 (8)
C54	0.0426 (12)	0.0287 (10)	0.0305 (10)	−0.0024 (9)	0.0055 (9)	0.0012 (8)
C55A	0.0313 (10)	0.0373 (11)	0.0395 (11)	−0.0147 (9)	−0.0010 (8)	−0.0001 (9)
C56	0.0365 (17)	0.0366 (19)	0.0347 (16)	−0.0110 (14)	0.0108 (13)	−0.0014 (13)
C57	0.047 (2)	0.067 (3)	0.0311 (17)	−0.029 (2)	0.0061 (15)	−0.0055 (17)
C55B	0.0313 (10)	0.0373 (11)	0.0395 (11)	−0.0147 (9)	−0.0010 (8)	−0.0001 (9)
C58	0.037 (4)	0.034 (4)	0.035 (3)	−0.012 (3)	0.008 (3)	0.002 (3)
C59	0.038 (4)	0.040 (4)	0.033 (3)	−0.011 (3)	−0.001 (3)	−0.005 (3)
C60	0.0295 (9)	0.0281 (9)	0.0330 (10)	−0.0043 (7)	0.0007 (7)	−0.0062 (7)
C61	0.0499 (15)	0.0497 (15)	0.0320 (11)	0.0018 (12)	0.0068 (10)	−0.0096 (10)
C62	0.0429 (13)	0.0337 (11)	0.0338 (11)	0.0020 (9)	−0.0019 (9)	0.0013 (9)
C63	0.0359 (11)	0.0309 (10)	0.0383 (11)	0.0019 (9)	0.0004 (9)	−0.0039 (8)
C64A	0.0387 (12)	0.0400 (12)	0.0390 (11)	−0.0154 (10)	0.0065 (9)	−0.0116 (9)
C65	0.0312 (15)	0.0344 (16)	0.0406 (16)	−0.0092 (12)	0.0085 (11)	−0.0078 (12)
C66	0.049 (2)	0.049 (2)	0.072 (3)	−0.0312 (19)	0.022 (2)	−0.023 (2)
C64B	0.0387 (12)	0.0400 (12)	0.0390 (11)	−0.0154 (10)	0.0065 (9)	−0.0116 (9)
C67	0.050 (7)	0.072 (10)	0.119 (12)	−0.024 (7)	0.039 (7)	−0.060 (8)
C68	0.074 (13)	0.15 (3)	0.125 (18)	−0.078 (16)	0.033 (10)	−0.050 (17)

### Geometric parameters (Å, º)

**Table:**

Cd1—N1	2.2273 (15)	C41—C42	1.379 (9)
Cd1—N4	2.2289 (16)	C41—H41	0.9500
Cd1—N3	2.2333 (16)	C42—C43	1.358 (8)
Cd1—N2	2.2440 (15)	C42—H42	0.9500
Cd1—Cl1	2.4847 (5)	C43—C44	1.396 (6)
N1—C4	1.369 (2)	C43—H43	0.9500
N1—C1	1.370 (2)	C44—H44	0.9500
N2—C6	1.367 (2)	C45—C50	1.367 (13)
N2—C9	1.371 (2)	C45—C46	1.394 (17)
N3—C11	1.367 (2)	C46—C47	1.395 (11)
N3—C14	1.367 (2)	C46—H46	0.9500
N4—C16	1.366 (2)	C47—C48	1.378 (15)
N4—C19	1.367 (2)	C47—H47	0.9500
C1—C20	1.413 (3)	C48—C49	1.348 (14)
C1—C2	1.447 (3)	C48—H48	0.9500
C2—C3	1.358 (3)	C49—C50	1.414 (12)
C2—H2	0.9500	C49—H49	0.9500
C3—C4	1.451 (3)	C50—H50	0.9500
C3—H3	0.9500	Ag2—C51	2.075 (2)
C4—C5	1.419 (3)	Ag2—C60	2.076 (2)
C5—C6	1.419 (3)	N5—C51	1.354 (3)
C5—C27	1.500 (3)	N5—C53	1.380 (3)
C6—C7	1.446 (3)	N5—C52	1.462 (3)
C7—C8	1.365 (3)	N6—C51	1.353 (3)
C7—H7	0.9500	N6—C54	1.369 (3)
C8—C9	1.446 (3)	N6—C55B	1.473 (3)
C8—H8	0.9500	N6—C55A	1.473 (3)
C9—C10	1.411 (3)	N7—C60	1.350 (3)
C10—C11	1.415 (3)	N7—C62	1.382 (3)
C10—C33	1.498 (2)	N7—C61	1.461 (3)
C11—C12	1.448 (3)	N8—C60	1.356 (3)
C12—C13	1.358 (3)	N8—C63	1.379 (3)
C12—H12	0.9500	N8—C64B	1.487 (3)
C13—C14	1.450 (3)	N8—C64A	1.487 (3)
C13—H13	0.9500	C52—H52A	0.9800
C14—C15	1.413 (3)	C52—H52B	0.9800
C15—C16	1.412 (3)	C52—H52C	0.9800
C15—C39	1.502 (7)	C53—C54	1.347 (4)
C15—C45	1.516 (17)	C53—H53	0.9500
C16—C17	1.448 (3)	C54—H54	0.9500
C17—C18	1.357 (3)	C55A—C56	1.566 (5)
C17—H17	0.9500	C55A—H55A	0.9900
C18—C19	1.453 (3)	C55A—H55B	0.9900
C18—H18	0.9500	C56—C57	1.514 (6)
C19—C20	1.414 (3)	C56—H56A	0.9900
C20—C21	1.495 (3)	C56—H56B	0.9900
C21—C26	1.392 (3)	C57—H57A	0.9800
C21—C22	1.394 (3)	C57—H57B	0.9800
C22—C23	1.394 (3)	C57—H57C	0.9800
C22—H22	0.9500	C55B—C58	1.452 (8)
C23—C24	1.380 (4)	C55B—H55C	0.9900
C23—H23	0.9500	C55B—H55D	0.9900
C24—C25	1.384 (4)	C58—C59	1.518 (12)
C24—H24	0.9500	C58—H58A	0.9900
C25—C26	1.392 (3)	C58—H58B	0.9900
C25—H25	0.9500	C59—H59A	0.9800
C26—H26	0.9500	C59—H59B	0.9800
C27—C32	1.393 (3)	C59—H59C	0.9800
C27—C28	1.395 (3)	C61—H61A	0.9800
C28—C29	1.395 (3)	C61—H61B	0.9800
C28—H28	0.9500	C61—H61C	0.9800
C29—C30	1.383 (4)	C62—C63	1.346 (4)
C29—H29	0.9500	C62—H62	0.9500
C30—C31	1.379 (4)	C63—H63	0.9500
C30—H30	0.9500	C64A—C65	1.521 (4)
C31—C32	1.396 (3)	C64A—H64A	0.9900
C31—H31	0.9500	C64A—H64B	0.9900
C32—H32	0.9500	C65—C66	1.539 (5)
C33—C38	1.382 (3)	C65—H65A	0.9900
C33—C34	1.387 (3)	C65—H65B	0.9900
C34—C35	1.391 (3)	C66—H66A	0.9800
C34—H34	0.9500	C66—H66B	0.9800
C35—C36	1.370 (4)	C66—H66C	0.9800
C35—H35	0.9500	C64B—C67	1.380 (18)
C36—C37	1.377 (4)	C64B—H64C	0.9900
C36—H36	0.9500	C64B—H64D	0.9900
C37—C38	1.394 (3)	C67—C68	1.20 (4)
C37—H37	0.9500	C67—H67A	0.9900
C38—H38	0.9500	C67—H67B	0.9900
C39—C44	1.378 (7)	C68—H68A	0.9800
C39—C40	1.403 (9)	C68—H68B	0.9800
C40—C41	1.401 (6)	C68—H68C	0.9800
C40—H40	0.9500		
			
N1—Cd1—N4	83.78 (6)	C43—C42—C41	120.3 (5)
N1—Cd1—N3	140.24 (6)	C43—C42—H42	119.8
N4—Cd1—N3	82.65 (6)	C41—C42—H42	119.8
N1—Cd1—N2	82.44 (6)	C42—C43—C44	120.7 (5)
N4—Cd1—N2	138.19 (6)	C42—C43—H43	119.7
N3—Cd1—N2	83.25 (6)	C44—C43—H43	119.7
N1—Cd1—Cl1	110.89 (4)	C39—C44—C43	120.5 (5)
N4—Cd1—Cl1	114.15 (5)	C39—C44—H44	119.8
N3—Cd1—Cl1	108.78 (5)	C43—C44—H44	119.8
N2—Cd1—Cl1	107.65 (5)	C50—C45—C46	118.3 (15)
C4—N1—C1	107.80 (15)	C50—C45—C15	118.6 (11)
C4—N1—Cd1	126.55 (12)	C46—C45—C15	120.0 (15)
C1—N1—Cd1	124.81 (12)	C45—C46—C47	118.7 (12)
C6—N2—C9	108.04 (15)	C45—C46—H46	120.6
C6—N2—Cd1	124.43 (12)	C47—C46—H46	120.6
C9—N2—Cd1	121.99 (12)	C48—C47—C46	121.0 (11)
C11—N3—C14	107.76 (15)	C48—C47—H47	119.5
C11—N3—Cd1	124.51 (12)	C46—C47—H47	119.5
C14—N3—Cd1	125.73 (12)	C49—C48—C47	119.0 (12)
C16—N4—C19	108.05 (16)	C49—C48—H48	120.5
C16—N4—Cd1	124.54 (13)	C47—C48—H48	120.5
C19—N4—Cd1	123.88 (13)	C48—C49—C50	120.6 (12)
N1—C1—C20	125.89 (17)	C48—C49—H49	119.7
N1—C1—C2	109.09 (16)	C50—C49—H49	119.7
C20—C1—C2	124.85 (17)	C45—C50—C49	119.3 (12)
C3—C2—C1	107.07 (17)	C45—C50—H50	120.3
C3—C2—H2	126.5	C49—C50—H50	120.3
C1—C2—H2	126.5	C51—Ag2—C60	179.14 (9)
C2—C3—C4	107.26 (17)	C51—N5—C53	110.6 (2)
C2—C3—H3	126.4	C51—N5—C52	125.1 (2)
C4—C3—H3	126.4	C53—N5—C52	124.3 (2)
N1—C4—C5	125.81 (16)	C51—N6—C54	111.3 (2)
N1—C4—C3	108.78 (16)	C51—N6—C55B	125.16 (19)
C5—C4—C3	125.36 (17)	C54—N6—C55B	123.4 (2)
C6—C5—C4	126.01 (16)	C51—N6—C55A	125.16 (19)
C6—C5—C27	117.04 (16)	C54—N6—C55A	123.4 (2)
C4—C5—C27	116.93 (16)	C60—N7—C62	111.3 (2)
N2—C6—C5	125.40 (16)	C60—N7—C61	124.5 (2)
N2—C6—C7	108.75 (16)	C62—N7—C61	124.1 (2)
C5—C6—C7	125.85 (16)	C60—N8—C63	111.3 (2)
C8—C7—C6	107.37 (16)	C60—N8—C64B	124.8 (2)
C8—C7—H7	126.3	C63—N8—C64B	123.8 (2)
C6—C7—H7	126.3	C60—N8—C64A	124.8 (2)
C7—C8—C9	106.83 (16)	C63—N8—C64A	123.8 (2)
C7—C8—H8	126.6	N6—C51—N5	104.55 (19)
C9—C8—H8	126.6	N6—C51—Ag2	125.11 (17)
N2—C9—C10	126.12 (16)	N5—C51—Ag2	130.24 (17)
N2—C9—C8	109.00 (16)	N5—C52—H52A	109.5
C10—C9—C8	124.85 (16)	N5—C52—H52B	109.5
C9—C10—C11	126.81 (16)	H52A—C52—H52B	109.5
C9—C10—C33	117.40 (16)	N5—C52—H52C	109.5
C11—C10—C33	115.74 (16)	H52A—C52—H52C	109.5
N3—C11—C10	125.60 (16)	H52B—C52—H52C	109.5
N3—C11—C12	109.05 (16)	C54—C53—N5	106.9 (2)
C10—C11—C12	125.32 (17)	C54—C53—H53	126.6
C13—C12—C11	107.16 (17)	N5—C53—H53	126.6
C13—C12—H12	126.4	C53—C54—N6	106.7 (2)
C11—C12—H12	126.4	C53—C54—H54	126.7
C12—C13—C14	106.97 (17)	N6—C54—H54	126.7
C12—C13—H13	126.5	N6—C55A—C56	110.5 (2)
C14—C13—H13	126.5	N6—C55A—H55A	109.6
N3—C14—C15	125.39 (17)	C56—C55A—H55A	109.6
N3—C14—C13	109.05 (16)	N6—C55A—H55B	109.6
C15—C14—C13	125.54 (17)	C56—C55A—H55B	109.6
C16—C15—C14	126.43 (17)	H55A—C55A—H55B	108.1
C16—C15—C39	116.5 (7)	C57—C56—C55A	112.8 (3)
C14—C15—C39	116.9 (7)	C57—C56—H56A	109.0
C16—C15—C45	119.1 (17)	C55A—C56—H56A	109.0
C14—C15—C45	114.4 (17)	C57—C56—H56B	109.0
N4—C16—C15	125.92 (17)	C55A—C56—H56B	109.0
N4—C16—C17	108.93 (17)	H56A—C56—H56B	107.8
C15—C16—C17	125.12 (18)	C56—C57—H57A	109.5
C18—C17—C16	107.21 (18)	C56—C57—H57B	109.5
C18—C17—H17	126.4	H57A—C57—H57B	109.5
C16—C17—H17	126.4	C56—C57—H57C	109.5
C17—C18—C19	107.02 (17)	H57A—C57—H57C	109.5
C17—C18—H18	126.5	H57B—C57—H57C	109.5
C19—C18—H18	126.5	C58—C55B—N6	113.6 (3)
N4—C19—C20	125.68 (17)	C58—C55B—H55C	108.8
N4—C19—C18	108.77 (17)	N6—C55B—H55C	108.8
C20—C19—C18	125.52 (17)	C58—C55B—H55D	108.8
C1—C20—C19	126.96 (16)	N6—C55B—H55D	108.8
C1—C20—C21	116.68 (17)	H55C—C55B—H55D	107.7
C19—C20—C21	116.23 (16)	C55B—C58—C59	110.2 (6)
C26—C21—C22	118.65 (18)	C55B—C58—H58A	109.6
C26—C21—C20	121.13 (18)	C59—C58—H58A	109.6
C22—C21—C20	120.21 (18)	C55B—C58—H58B	109.6
C21—C22—C23	120.3 (2)	C59—C58—H58B	109.6
C21—C22—H22	119.8	H58A—C58—H58B	108.1
C23—C22—H22	119.8	C58—C59—H59A	109.5
C24—C23—C22	120.4 (2)	C58—C59—H59B	109.5
C24—C23—H23	119.8	H59A—C59—H59B	109.5
C22—C23—H23	119.8	C58—C59—H59C	109.5
C23—C24—C25	119.8 (2)	H59A—C59—H59C	109.5
C23—C24—H24	120.1	H59B—C59—H59C	109.5
C25—C24—H24	120.1	N7—C60—N8	104.14 (19)
C24—C25—C26	119.9 (2)	N7—C60—Ag2	128.48 (16)
C24—C25—H25	120.0	N8—C60—Ag2	127.32 (17)
C26—C25—H25	120.0	N7—C61—H61A	109.5
C21—C26—C25	120.8 (2)	N7—C61—H61B	109.5
C21—C26—H26	119.6	H61A—C61—H61B	109.5
C25—C26—H26	119.6	N7—C61—H61C	109.5
C32—C27—C28	117.72 (18)	H61A—C61—H61C	109.5
C32—C27—C5	121.56 (18)	H61B—C61—H61C	109.5
C28—C27—C5	120.72 (18)	C63—C62—N7	106.7 (2)
C27—C28—C29	121.0 (2)	C63—C62—H62	126.7
C27—C28—H28	119.5	N7—C62—H62	126.7
C29—C28—H28	119.5	C62—C63—N8	106.5 (2)
C30—C29—C28	120.5 (2)	C62—C63—H63	126.7
C30—C29—H29	119.8	N8—C63—H63	126.7
C28—C29—H29	119.8	N8—C64A—C65	113.2 (2)
C31—C30—C29	119.1 (2)	N8—C64A—H64A	108.9
C31—C30—H30	120.4	C65—C64A—H64A	108.9
C29—C30—H30	120.4	N8—C64A—H64B	108.9
C30—C31—C32	120.6 (2)	C65—C64A—H64B	108.9
C30—C31—H31	119.7	H64A—C64A—H64B	107.8
C32—C31—H31	119.7	C64A—C65—C66	114.0 (3)
C27—C32—C31	121.1 (2)	C64A—C65—H65A	108.8
C27—C32—H32	119.5	C66—C65—H65A	108.8
C31—C32—H32	119.5	C64A—C65—H65B	108.8
C38—C33—C34	117.84 (18)	C66—C65—H65B	108.8
C38—C33—C10	120.88 (18)	H65A—C65—H65B	107.7
C34—C33—C10	121.26 (18)	C65—C66—H66A	109.5
C33—C34—C35	121.1 (2)	C65—C66—H66B	109.5
C33—C34—H34	119.4	H66A—C66—H66B	109.5
C35—C34—H34	119.4	C65—C66—H66C	109.5
C36—C35—C34	120.4 (2)	H66A—C66—H66C	109.5
C36—C35—H35	119.8	H66B—C66—H66C	109.5
C34—C35—H35	119.8	C67—C64B—N8	101.6 (10)
C35—C36—C37	119.2 (2)	C67—C64B—H64C	111.5
C35—C36—H36	120.4	N8—C64B—H64C	111.4
C37—C36—H36	120.4	C67—C64B—H64D	111.4
C36—C37—C38	120.4 (2)	N8—C64B—H64D	111.4
C36—C37—H37	119.8	H64C—C64B—H64D	109.3
C38—C37—H37	119.8	C68—C67—C64B	135 (3)
C33—C38—C37	121.0 (2)	C68—C67—H67A	103.4
C33—C38—H38	119.5	C64B—C67—H67A	103.4
C37—C38—H38	119.5	C68—C67—H67B	103.4
C44—C39—C40	118.6 (6)	C64B—C67—H67B	103.4
C44—C39—C15	122.3 (5)	H67A—C67—H67B	105.2
C40—C39—C15	118.8 (6)	C67—C68—H68A	109.5
C41—C40—C39	120.2 (5)	C67—C68—H68B	109.5
C41—C40—H40	119.9	H68A—C68—H68B	109.5
C39—C40—H40	119.9	C67—C68—H68C	109.5
C42—C41—C40	119.6 (5)	H68A—C68—H68C	109.5
C42—C41—H41	120.2	H68B—C68—H68C	109.5
C40—C41—H41	120.2		
			
C4—N1—C1—C20	−175.51 (19)	C23—C24—C25—C26	0.2 (4)
Cd1—N1—C1—C20	14.4 (3)	C22—C21—C26—C25	−0.6 (3)
C4—N1—C1—C2	−0.1 (2)	C20—C21—C26—C25	177.9 (2)
Cd1—N1—C1—C2	−170.14 (13)	C24—C25—C26—C21	0.2 (4)
N1—C1—C2—C3	0.1 (2)	C6—C5—C27—C32	−58.8 (3)
C20—C1—C2—C3	175.6 (2)	C4—C5—C27—C32	119.8 (2)
C1—C2—C3—C4	−0.1 (2)	C6—C5—C27—C28	122.1 (2)
C1—N1—C4—C5	177.60 (19)	C4—C5—C27—C28	−59.3 (3)
Cd1—N1—C4—C5	−12.6 (3)	C32—C27—C28—C29	0.6 (3)
C1—N1—C4—C3	0.0 (2)	C5—C27—C28—C29	179.8 (2)
Cd1—N1—C4—C3	169.83 (13)	C27—C28—C29—C30	−0.7 (4)
C2—C3—C4—N1	0.1 (2)	C28—C29—C30—C31	0.1 (4)
C2—C3—C4—C5	−177.5 (2)	C29—C30—C31—C32	0.6 (4)
N1—C4—C5—C6	−6.6 (3)	C28—C27—C32—C31	0.1 (3)
C3—C4—C5—C6	170.6 (2)	C5—C27—C32—C31	−179.05 (19)
N1—C4—C5—C27	174.96 (18)	C30—C31—C32—C27	−0.7 (3)
C3—C4—C5—C27	−7.8 (3)	C9—C10—C33—C38	78.7 (3)
C9—N2—C6—C5	−178.04 (18)	C11—C10—C33—C38	−99.0 (2)
Cd1—N2—C6—C5	28.0 (3)	C9—C10—C33—C34	−102.9 (3)
C9—N2—C6—C7	0.9 (2)	C11—C10—C33—C34	79.4 (3)
Cd1—N2—C6—C7	−153.07 (14)	C38—C33—C34—C35	0.1 (4)
C4—C5—C6—N2	−2.0 (3)	C10—C33—C34—C35	−178.4 (3)
C27—C5—C6—N2	176.36 (18)	C33—C34—C35—C36	0.6 (5)
C4—C5—C6—C7	179.2 (2)	C34—C35—C36—C37	0.2 (5)
C27—C5—C6—C7	−2.4 (3)	C35—C36—C37—C38	−1.7 (5)
N2—C6—C7—C8	−0.7 (2)	C34—C33—C38—C37	−1.6 (4)
C5—C6—C7—C8	178.22 (19)	C10—C33—C38—C37	176.9 (3)
C6—C7—C8—C9	0.3 (2)	C36—C37—C38—C33	2.4 (5)
C6—N2—C9—C10	177.15 (19)	C16—C15—C39—C44	−72.0 (13)
Cd1—N2—C9—C10	−28.1 (3)	C14—C15—C39—C44	104.5 (13)
C6—N2—C9—C8	−0.8 (2)	C16—C15—C39—C40	115.1 (11)
Cd1—N2—C9—C8	153.99 (14)	C14—C15—C39—C40	−68.3 (12)
C7—C8—C9—N2	0.3 (2)	C44—C39—C40—C41	4.4 (17)
C7—C8—C9—C10	−177.6 (2)	C15—C39—C40—C41	177.5 (8)
N2—C9—C10—C11	0.2 (3)	C39—C40—C41—C42	−1.5 (13)
C8—C9—C10—C11	177.8 (2)	C40—C41—C42—C43	−1.3 (12)
N2—C9—C10—C33	−177.15 (18)	C41—C42—C43—C44	1.1 (11)
C8—C9—C10—C33	0.5 (3)	C40—C39—C44—C43	−4.6 (16)
C14—N3—C11—C10	−178.68 (19)	C15—C39—C44—C43	−177.4 (8)
Cd1—N3—C11—C10	16.6 (3)	C42—C43—C44—C39	1.9 (11)
C14—N3—C11—C12	−0.3 (2)	C16—C15—C45—C50	−90 (3)
Cd1—N3—C11—C12	−164.99 (13)	C14—C15—C45—C50	93 (3)
C9—C10—C11—N3	6.5 (3)	C16—C15—C45—C46	69 (3)
C33—C10—C11—N3	−176.04 (18)	C14—C15—C45—C46	−107 (3)
C9—C10—C11—C12	−171.6 (2)	C50—C45—C46—C47	−17 (4)
C33—C10—C11—C12	5.8 (3)	C15—C45—C46—C47	−177 (2)
N3—C11—C12—C13	0.1 (2)	C45—C46—C47—C48	4 (3)
C10—C11—C12—C13	178.5 (2)	C46—C47—C48—C49	8 (3)
C11—C12—C13—C14	0.1 (2)	C47—C48—C49—C50	−6 (3)
C11—N3—C14—C15	178.69 (19)	C46—C45—C50—C49	20 (4)
Cd1—N3—C14—C15	−16.9 (3)	C15—C45—C50—C49	180 (2)
C11—N3—C14—C13	0.4 (2)	C48—C49—C50—C45	−8 (3)
Cd1—N3—C14—C13	164.82 (14)	C54—N6—C51—N5	−0.9 (3)
C12—C13—C14—N3	−0.3 (2)	C55B—N6—C51—N5	174.7 (2)
C12—C13—C14—C15	−178.6 (2)	C55A—N6—C51—N5	174.7 (2)
N3—C14—C15—C16	−5.2 (3)	C54—N6—C51—Ag2	175.85 (17)
C13—C14—C15—C16	172.9 (2)	C55B—N6—C51—Ag2	−8.5 (3)
N3—C14—C15—C39	178.7 (3)	C55A—N6—C51—Ag2	−8.5 (3)
C13—C14—C15—C39	−3.3 (4)	C53—N5—C51—N6	0.6 (3)
N3—C14—C15—C45	171.2 (7)	C52—N5—C51—N6	179.1 (2)
C13—C14—C15—C45	−10.8 (7)	C53—N5—C51—Ag2	−175.94 (18)
C19—N4—C16—C15	−176.8 (2)	C52—N5—C51—Ag2	2.6 (3)
Cd1—N4—C16—C15	23.8 (3)	C51—N5—C53—C54	−0.1 (3)
C19—N4—C16—C17	1.2 (2)	C52—N5—C53—C54	−178.6 (2)
Cd1—N4—C16—C17	−158.20 (15)	N5—C53—C54—N6	−0.5 (3)
C14—C15—C16—N4	1.3 (4)	C51—N6—C54—C53	0.9 (3)
C39—C15—C16—N4	177.5 (3)	C55B—N6—C54—C53	−174.8 (2)
C45—C15—C16—N4	−174.9 (7)	C55A—N6—C54—C53	−174.8 (2)
C14—C15—C16—C17	−176.4 (2)	C51—N6—C55A—C56	−102.6 (3)
C39—C15—C16—C17	−0.2 (4)	C54—N6—C55A—C56	72.5 (3)
C45—C15—C16—C17	7.4 (8)	N6—C55A—C56—C57	62.4 (4)
N4—C16—C17—C18	−0.9 (3)	C51—N6—C55B—C58	−65.1 (4)
C15—C16—C17—C18	177.1 (2)	C54—N6—C55B—C58	110.1 (4)
C16—C17—C18—C19	0.3 (3)	N6—C55B—C58—C59	−61.8 (6)
C16—N4—C19—C20	176.82 (19)	C62—N7—C60—N8	−0.9 (3)
Cd1—N4—C19—C20	−23.6 (3)	C61—N7—C60—N8	176.4 (3)
C16—N4—C19—C18	−1.0 (2)	C62—N7—C60—Ag2	176.5 (2)
Cd1—N4—C19—C18	158.55 (14)	C61—N7—C60—Ag2	−6.2 (4)
C17—C18—C19—N4	0.4 (3)	C63—N8—C60—N7	1.5 (3)
C17—C18—C19—C20	−177.4 (2)	C64B—N8—C60—N7	−175.5 (2)
N1—C1—C20—C19	7.1 (3)	C64A—N8—C60—N7	−175.5 (2)
C2—C1—C20—C19	−167.7 (2)	C63—N8—C60—Ag2	−175.95 (19)
N1—C1—C20—C21	−177.19 (18)	C64B—N8—C60—Ag2	7.0 (4)
C2—C1—C20—C21	8.1 (3)	C64A—N8—C60—Ag2	7.0 (4)
N4—C19—C20—C1	−1.9 (3)	C60—N7—C62—C63	0.0 (3)
C18—C19—C20—C1	175.6 (2)	C61—N7—C62—C63	−177.3 (3)
N4—C19—C20—C21	−177.64 (19)	N7—C62—C63—N8	0.9 (3)
C18—C19—C20—C21	−0.1 (3)	C60—N8—C63—C62	−1.6 (3)
C1—C20—C21—C26	71.7 (3)	C64B—N8—C63—C62	175.5 (2)
C19—C20—C21—C26	−112.1 (2)	C64A—N8—C63—C62	175.5 (2)
C1—C20—C21—C22	−109.8 (2)	C60—N8—C64A—C65	−102.4 (3)
C19—C20—C21—C22	66.4 (3)	C63—N8—C64A—C65	81.0 (3)
C26—C21—C22—C23	0.5 (3)	N8—C64A—C65—C66	65.4 (4)
C20—C21—C22—C23	−178.0 (2)	C60—N8—C64B—C67	−74.2 (10)
C21—C22—C23—C24	0.0 (4)	C63—N8—C64B—C67	109.2 (10)
C22—C23—C24—C25	−0.3 (4)	N8—C64B—C67—C68	−44 (3)

## References

[bb1] Achar, G., Shahini, C. R., Patil, S. A. & Budagumpi, S. (2017). *J. Organomet. Chem.* **833**, 28–42.

[bb2] Bruker (2003). *SAINT*. Bruker AXS Inc., Madison, Wisconsin, USA.

[bb3] Bruker (2017). *APEX3*. Bruker AXS Inc., Madison, Wisconsin, USA.

[bb4] Byrn, M. P., Curtis, C. J., Goldberg, I., Hsiou, Y., Khan, S. I., Sawin, P. A., Tendick, S. K. & Strouse, E. C. (1991). *J. Am. Chem. Soc.* **126**, 6549–6557.

[bb5] Hübschle, C. B., Sheldrick, G. M. & Dittrich, B. (2011). *J. Appl. Cryst.* **44**, 1281–1284. 10.1107/S0021889811043202 PMC3246833 22477785

[bb6] Krause, L., Herbst-Irmer, R., Sheldrick, G. M. & Stalke, D. (2015). *J. Appl. Cryst.* **48**, 3–10. 10.1107/S1600576714022985 PMC4453166 26089746

[bb7] Sheldrick, G. M. (2015*a*). *Acta Cryst.* A**71**, 3–8.

[bb8] Sheldrick, G. M. (2015*b*). *Acta Cryst.* A**71**, 3–8.

[bb9] Spek, A. L. (2020). *Acta Cryst.* E**76**, 1–11. 10.1107/S2056989019016244 PMC6944088 31921444

[bb10] Toumi, H., Belghith, Y., Daran, J.-C. & Nasri, H. (2013). *Acta Cryst.* E**69**, m354–m355. 10.1107/S160053681301489X PMC3772402 24046545

[bb11] Zhang, Z., Gao, W.-Y., Wojtas, L., Ma, S., Eddaoudi, M. & Zaworotko, M. J. (2012). *Angew. Chem. Int. Ed.* **51**, 9330–9334. 10.1002/anie.201203594 22907717

[bb12] Zhang, Z., Zhang, L., Wojtas, L., Nugent, P., Eddaoudi, M. & Zaworotko, M. J. (2012). *J. Am. Chem. Soc.* **134**, 924–927. 10.1021/ja209643b 22191602

